# A mesophotic black coral forest in the Adriatic Sea

**DOI:** 10.1038/s41598-020-65266-9

**Published:** 2020-05-22

**Authors:** Giovanni Chimienti, Diana De Padova, Michele Mossa, Francesco Mastrototaro

**Affiliations:** 1 Department of Biology, University of Bari Aldo Moro, Bari, Italy; 2 CoNISMa, Roma, Italy; 3 Polytechnic University of Bari, DICATECh, Bari, Italy

**Keywords:** Marine biology, Biodiversity, Conservation biology, Zoology

## Abstract

A forest of the black coral *Antipathella subpinnata* was found from 52 to 80 m depth in three different sites at Tremiti Islands Marine Protected Area (MPA; Mediterranean Sea), with two of them hosting a monospecific forest on horizontal and vertical substrates. Colonies of *A. subpinnata* showed a mean density between 0.22 ± 0.03 and 2.40 ± 0.26 colonies m^−2^ (maximum local values of 2.4–7.2 colonies m^−2^). The link between the local distribution of *A. subpinnata* and the main oceanographic features confirmed the fundamental role of the currents in shaping the distribution of the species in presence of hard substrata. This black coral forest represents the only one known thus far in the Adriatic Sea, but it could be linked with other unseen forests all over the Mediterranean Sea. The associated megafauna highlights the importance of these forests as habitat for species of both conservation and commercial importance but, at the same time, makes such habitat a target for fishing practices, as many lost fishing gears were found within the coral forest. The enlargement of the MPA borders and the enforcement of controls in the area of the *A. subpinnata* forest is urgently needed for the proper conservation of this protected species.

## Introduction

The order Antipatharia groups all those colonial hexacorals commonly known as black corals, with representatives from tropical to polar ecosystems displaying a wide range of bathymetric distribution, colony sizes and morphologies^1^. Their name comes from the Greek “*anti pàthos*”, literally “against suffering”, because these rare corals were used in the past for medical purposes or to make amulets against illnesses and, currently, some antipatharian species are still used in the jewellery industry. Black corals often live as isolated colonies but, under proper conditions, they can form dense aggregations that belong to the so-called “coral forests” or “coral gardens”^2–5^, representing Vulnerable Marine Ecosystems (VMEs). These forests can be monospecific or can consist of a combination of coral species constituting complex communities that can include both hexacorals and octocorals^6^. In the Mediterranean Sea, black coral forests are important three-dimensional habitats of the mesophotic and aphotic zones that host a rich associated fauna and act as spawning, nursery and feeding areas for numerous species^7^. Although data about the occurrence of black corals have exponentially increased in the last two decades thanks to the development of technical scuba diving techniques and the use of Remotely Operated Vehicles (ROVs), the occurrence of Mediterranean black coral forests is still rare and scantly known^6–11^. *Antipathella subpinnata* Ellis and Solander, 1786 is an Atlantic-Mediterranean species that can reach more than 1 m in height and represents a key habitat-former of the lower fringe of the mesophotic zone^8^. This species settles on hard substrata from 50 to 600 m depth, although it is mostly common within 200 m depth^8–10^. It is characterized by a branched corallum with simple elongated pseudopinnules arranged irregularly in 1–4 rows^1^. The remarkable ecological importance of *A. subpinnata* as habitat former has been widely recognized and the species has been listed as “near threatened” in the Red List by the International Union for Conservation of Nature (IUCN)^12^. *Antipathella* forests are currently designated as under threat and/or in decline in OSPAR Regions I, II, IV and V^13^ (Oslo/Paris Convention for the protection of Marine Environment of the North-East Atlantic), and their need for protection has been highlighted under several legal instruments for species conservation and management, such as Bern Convention (Appendix III), Barcelona Convention (Annex II and III; key species for the identification of Specially Protected Areas of Mediterranean Importance, SPAMI), and CITES (Convention on International Trade in Endangered Species of Wild Fauna and Flora). In the Mediterranean Sea, forests of *A. subpinnata* have been reported in: Northern Tyrrhenian Sea, with two forests of 0.27 ± 0.10 colonies m^−2^ (96 − 117 m depth) and of 0.13 ± 0.04 colonies m^−2^ (127 – 170 m depth), respectively^7^; Southern Tyrrhenian Sea, with a density of 1.4 colonies m^−2^ (maximum 5.2 colonies m^−2^; 55 − 80 m depth) in a mixed coral aggregation with the alcyonaceans *Paramuricea clavata* and *Eunicella cavolini*^9^; Sardinia Channel, with a mixed coral forest where *A. subpinnata* is not dominant (0.11 ± 0.06 colonies m^−2^; 120 − 170 m depth)^14^. Further unquantified forests have been reported as mixed coral assemblages in the Ligurian Sea (off Bordighera, 64–75 m depth; on the *Ravenna* wreck, 80–92 m depth), in the Tyrrhenian Sea (off Nisida Island, Gulf of Naples, ca. 100 m depth; Northern Sicily, 55–65 m depth) and in the Sicily Channel (Maltese Island, 250–400 m depth)^8,10,15^. In the Atlantic Ocean, a forest of *A. subpinnata* has been found off the Azores (150–196 m depth), with an average coral density of 0.75 ± 0.72 colonies m^−2^ (maximum density of 2.64 colonies m^−2^)^11^, while unquantified forests have also been reported along the coasts of Spain (Castro Verde Bank, Bay of Biscay and Villar de Fuentes, Rías Baixas) and Portugal (Saint Vincent Cape and Ormonde, Gorringe Bank)^16^.

Here we describe an extensive population of *A. subpinnata* found on the mesophotic rocky bottoms at Tremiti Islands Marine Protected Area (MPA), in the Adriatic Sea. The seabed below the depth of 50 m is in the proximity of the MPA border and has been scantly explored thus far, resulting in a substantial lack of knowledge about the animal communities of the mesophotic zone around the Tremiti Archipelago. In particular, *A. subpinnata* has been reported in the area with only one juvenile colony found at 51 m in depth^17^, that disappeared two years later for unknown causes.

## Results

### **The population of*Antipathella subpinnata*at Tremiti Archipelago**

**Figure 1:**
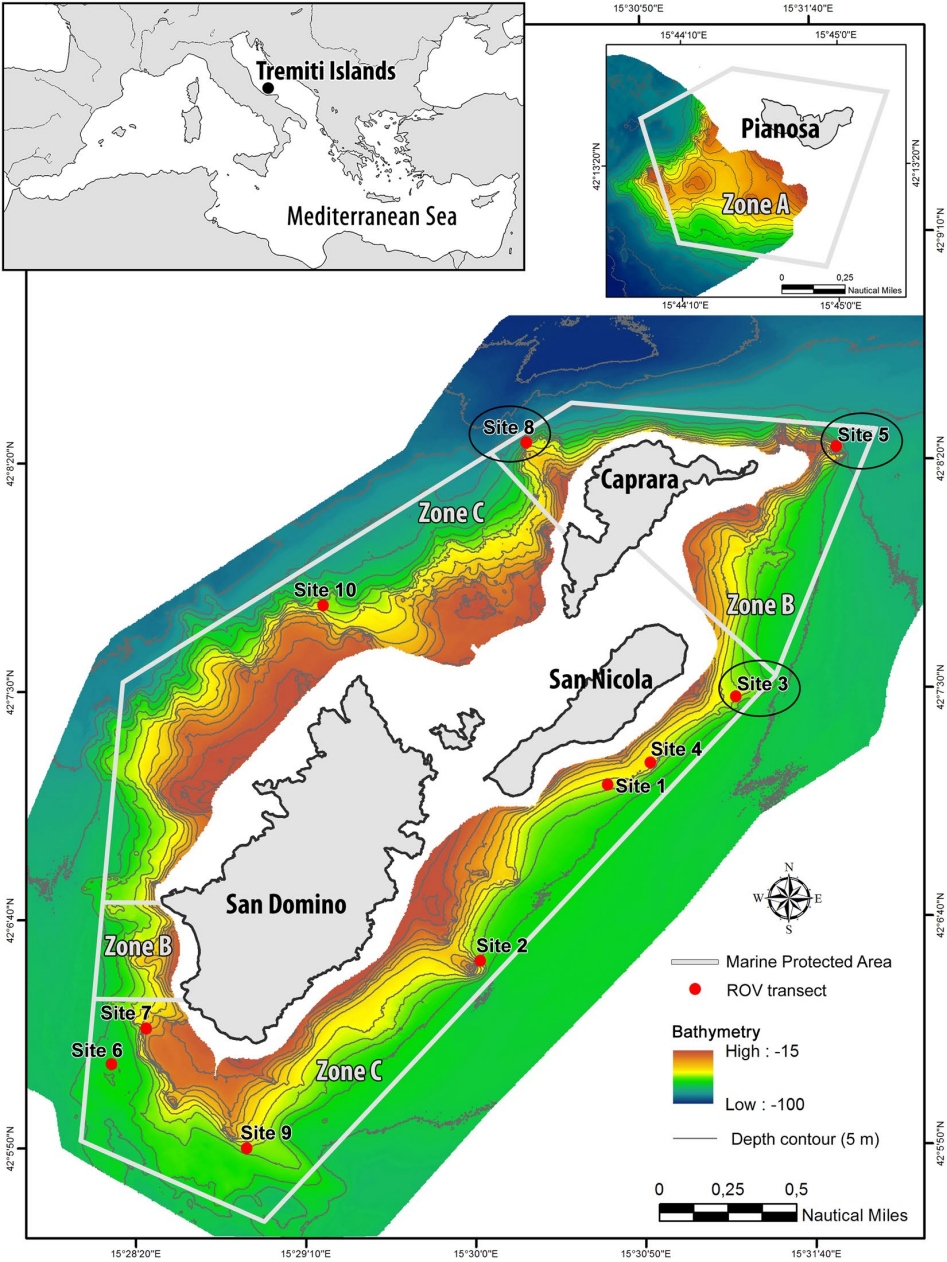
Study area with indication of bathymetry (from 15 to 100 m depth), Marine Protected Area zonation, location of the ROV transects and indication of the sites where a forest of *Antipathella subpinnata* was observed (black circle). Zone A: no take, no entry zone; Zone B: highly protected zone; Zone C: partially protected zone. Map has been created using ESRI ARCMAP 10.2 (https://support.esri.com/en/products/desktop/arcgis-desktop/arcmap/10-2-2) with DTM and image produced by CARIS SIPS 8 (https://www.teledynecaris.com/en/products/hips-and-sips/).

**Figure 2:**
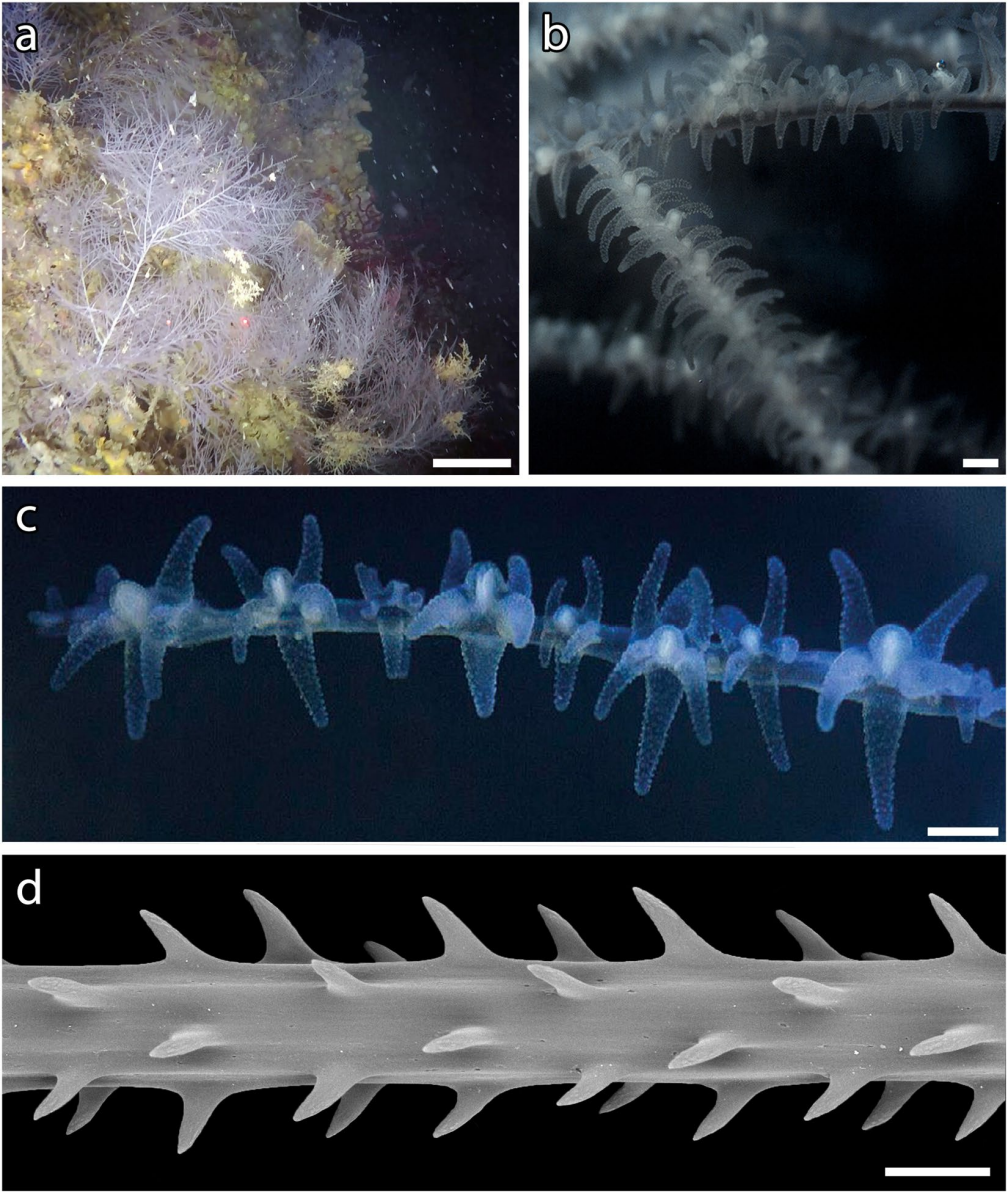
*Antipathella subpinnata*. (**a**) Colonies on sub-vertical substrate (scale bar: 20 cm); (**b**) branches and branchlets (scale bar: 1 mm); (**c**) polyps (scale bar: 1 mm); (**d**) spines on a terminal branch (scale bar: 100 μm).

A total of 839 colonies of *A. subpinnata* were observed from 52 to 80 m depth, with the sparse occurrence of isolated colonies and the presence of a forest in three different main sites in the northeast area of the archipelago (Fig. 1; Table S1). Colonies were arborescent, with a single stem from which numerous, long and flexible branches and branchlets took origin (Fig. 2a). Polyps on the ramifications were white and monoserially arranged (Fig. 2b–c). The skeleton of the branchlets was characterized by simple, triangular spines oriented upwards and 80–150 μm long (Fig. 2d).

Colonies were settled on animal-dominated bioconstructions characterized by corals, bryozoans, serpulids, molluscs, sponges and encrusting epifauna that, despite the scarce presence of calcareous red algae in the deepest part, could be considered a coralligenous habitat^18,19^. This habitat constituted hard-bottom oasis on the surrounding muddy bottom colonized by the sea pen *Pennatula rubra* and the sea cucumber *Holothuria (Holothuria) tubulosa*.

**Figure 3:**
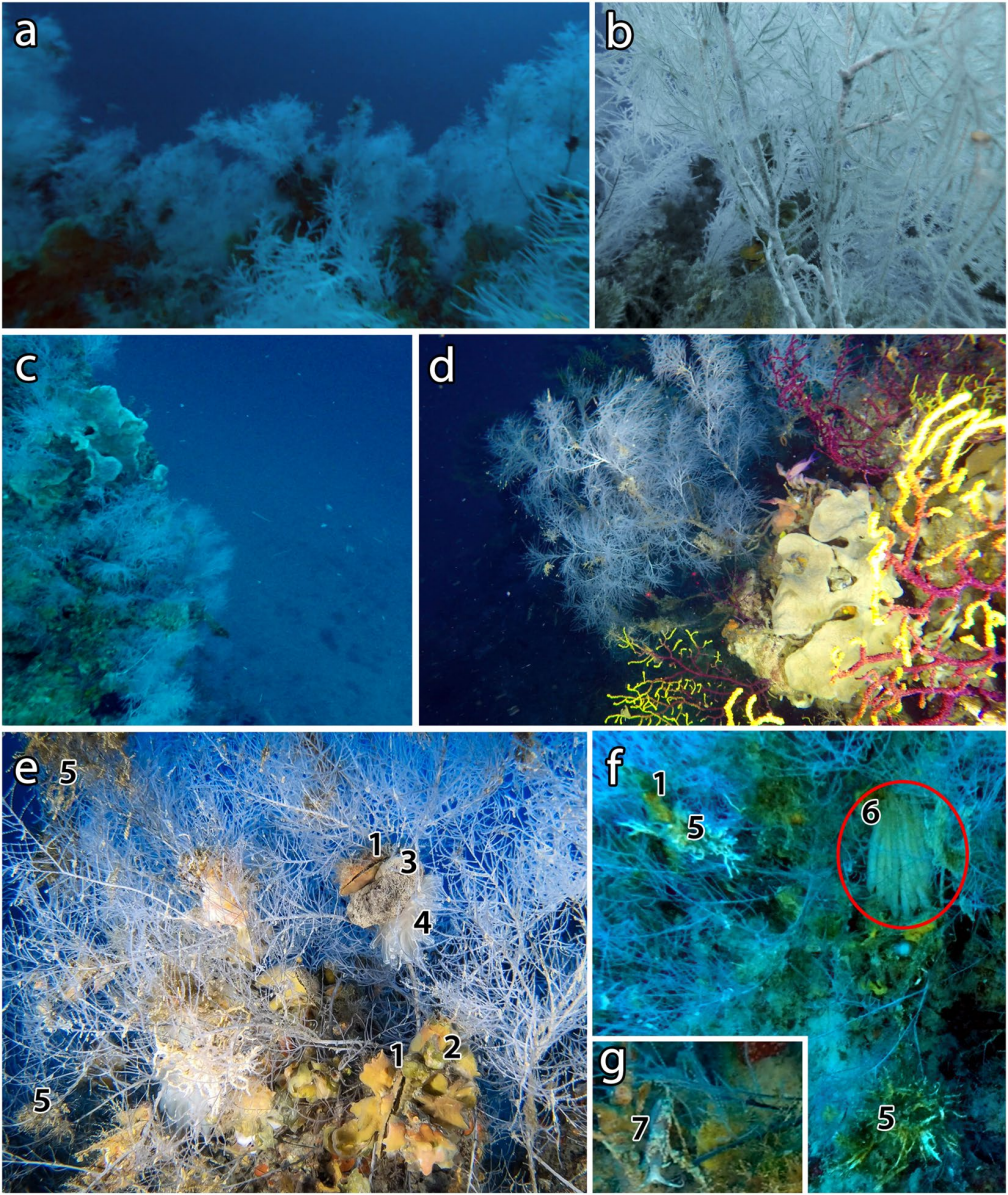
The forest of *Antipathella subpinnata* of Tremiti Islands. (**a**) Dense monospecific forest on horizontal substrate (site 3), with (**b**) close detail; (**c**) dense forest with large sponges and bryozoans on vertical substrate (site 5); (**d**) isolated colonies in mixed aggregation with *Paramuricea clavata* on sub-vertical substrate (site 8). Epibionts: (**e**) *Ostrea edulis* covered with hydroids (1), sponges and bryozoans (2), Didemnidae ascidians (3), *Clavelina lepadiformis* (4), *Filograna* sp. (5); (**f**) squid egg masses (6, red circle) laid on a colony of *A. subpinnata*; (**g**) Facelinidae nudibranch (7) on a colony of *A. subpinnata*.

The three sites characterized by the black coral forest showed different edaphic features (e.g. slope of the substratum, presence of shoals or scattered bioconstructions, orientation), colonies densities and composition of the benthic community that made them not comparable among each other. In detail, site 3 consisted of scattered rocky pinnacles where *A. subpinnata* was mostly settled on top (Fig. 3a–b), with a mean density of 2.40 ± 0.26 (79 sampling units; occupancy 77.21%) and a maximum of 7.2 colonies m^−2^. Site 5 was characterized by a large shoal where *A. subpinnata* formed a monospecific forest on a vertical wall (Fig. 3c) with a mean density of 1.17 ± 0.14 colonies m^−2^ (129 sampling units; occupancy 48.84%) and a maximum of 6.8 colonies m^−2^. Site 8 consisted of a shoal where *A. subpinnata* formed a mixed coral forest with *P. clavata* (Fig. 3d) characterized by a mean density of *A. subpinnata* of 0.22 ± 0.03 colonies m^−2^ (116 sampling units; occupancy 43.96%) and a maximum of 2.4 colonies m^−2^.

**Figure 4:**
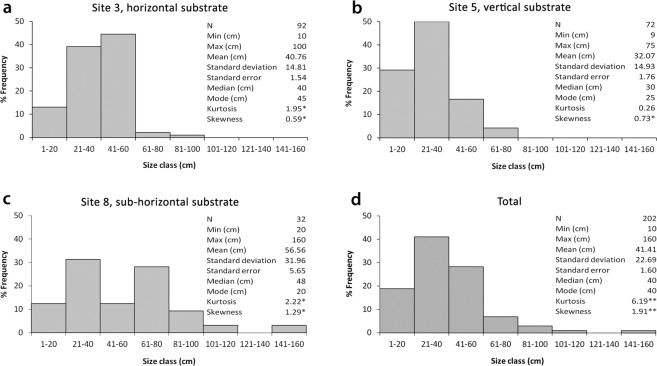
Size-structure distribution of *A. subpinnata* at Tremiti Archipelago. (**a**) Site 3; (**b**) site 5; (**c**) site 8; (**d**) considering the total colonies measured. Significance: *p < 0.05; **p < 0.001.

Size-frequency histograms for each site showed that most of the colonies were smaller than 60 cm (Fig. 4a–d). In particular, the sub-population of site 3 resulted mostly composed of colonies belonging to the size classes 21–40 and 41–60. Size–frequency distribution was mesokurtic, with a slightly positive skewness indicating the presence of a right tail represented by colonies higher than 60 cm (Fig. 4a). In site 5 the colonies were generally smaller, since 1–20 and 21–40 were the most represented size classes. The distribution was right-tailed and kurtosis resulted not significant in this case due to the absence of colonies higher than 80 cm (Fig. 4b). The most common size classes in site 8 were 21–40 and 61–80, with a mesokurtic distribution and a positive skewness indicating a right tail represented by colonies higher than 81–100 cm (Fig. 4c).

Considering all the colonies of *A. subpinnata* measured, including isolated colonies outside the main forest, the population of Tremiti Islands resulted mostly represented by colonies smaller than 60 cm, with a leptokurtic distribution and an evident right tail (Fig. 4d).

### Epibiosis and associated megafauna

**Figure 5:**
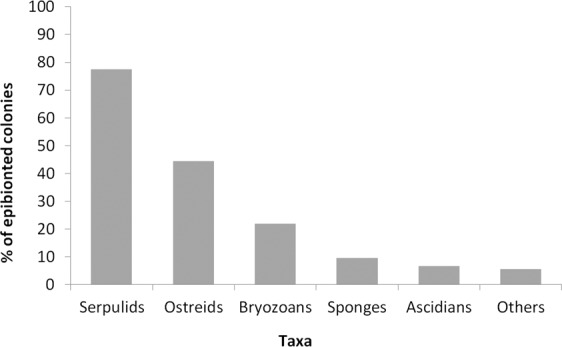
Main epibiont taxa on *Antipathella subpinnata*.

The 25.1% of colonies (211 out of 839) hosted epibionts, particularly serpulids of the genus *Filograna* and molluscs, mostly the ostreids *Pteria hirundo* and *Ostrea edulis*, settled on both living and dead parts of the colonies (Fig. 3e–f; Fig. 5). Unidentified bryozoans, sponges and ascidians were also present, mostly settled on dead branches as well as on the molluscs shells and the serpulids tubes. Other epibionts included hydrozoans on both *A. subpinnata* and on the valves of *O. edulis*, although it was not possible to quantify them because they were often indistinguishable from the white living branches of *A. subpinnata*. Erected brown macroalgae, identified as *Sporochnus* sp., were occasionally observed on colonies from 50 to 62 m depth. Squid egg masses were present on 1% of the colonies observed, attesting the use of these forests as a spawning area for species of commercial interest. Furthermore, two specimens of an unidentified nudibranch, likely belonging to Facelinidae family, were observed on the branches of two different colonies of *A. subpinnata* at site 3 (Fig. 3g).

**Figure 6:**
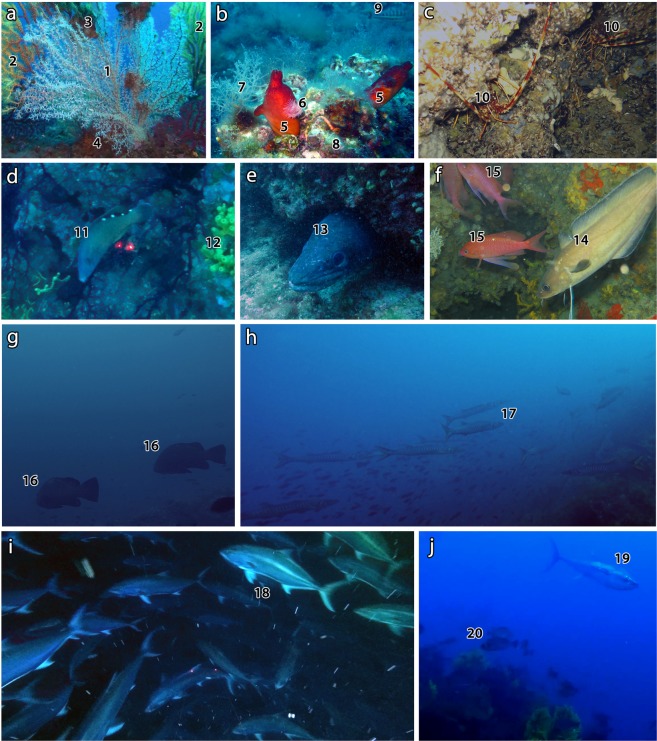
Megafauna associated with the forest of *A. subpinnata*. (**a**) the zoanthid *Savalia savaglia* (1) parasiting the red gorgonian *Paramuricea clavata* (2), with epibiont ostreids (3) and the bryozoan *Myriapora truncata* (4); (**b**) the tunicate *Halocynthia papillosa* (5), the serpulid *Serpula vermicularis* (6) and unidentified hydrozoans (7) settled on a coralligenous community made by encrusting fauna (8); in the back, the comber *Serranus cabrilla* (9); (**c**) the lobster *Palinurus elephas* (10); (**d**) reproductive female of the cuckoo wrasse *Labrus mixtus* (11) and the sponge *Aplysina aerophoba* (12); (**e**) the conger eel *Conger conger* (13); (**f**) the forkbeard *Phycis phycis* (14) and the swallowtail seaperch *Anthias anthias* (15); (**g**) the dusky grouper *Epinephelus marginatus* (16); (**h**) school of the yellowmouth barracuda *Sphyraena viridensis* (17); (**i**) hunting school of the greater amberjack *Seriola dumerili* (18); (**j**) the bluefin tuna *Thunnus thynnus* searching for preys (19) and a school of the black seabream *Spondyliosoma cantharus* (20) hiding finding refuge in the coral forest.

Associated megafauna observed within the coral forest included 68 taxa (11 Porifera, 5 Cnidaria, 8 Mollusca, 4 Annelida, 4 Crustacea, 4 Bryozoa, 7 Echinodermata, 3 Tunicata and 22 Pisces) (Table 1; Fig. 6a–j). Thirty-six of the taxa found are listed in at least one of the main legal instruments for species conservation and management ongoing in the Mediterranean Sea, such as the Bern Convention (Convention on the Conservation of European Wildlife and Natural Habitats: Appendix II: strictly protected fauna species; Appendix III: protected fauna species), the Barcelona Convention (Protocol for Specially Protected Areas and Biological Diversity in the Mediterranean, SPA/BD: Annex II: list of endangered or threatened species; Annex III: list of species whose exploitation is regulated), the IUCN Red List and the EU Habitat Directive (92/43/EEC; Annex IV: animal and plant species of community interest in need of strict protection) (Table 1).

**Table 1:** Animal taxa observed in the three sites characterized by the presence of a forest of *Antipathella subpinnata*, with indication of the main protection framework.

**Taxa**	**Authors**	**Site 3**	**Site 5**	**Site 8**	**Protection**		
					**B**	**S**	**I**
**Porifera**							
*Aplysina aerophoba*	(Nardo, 1833)		●	●		II	
*Axinella* spp.		●	●	●			
*Clathria* sp.		●	●				
*Dysidea* sp.		●	●	●			
*Haliclona* sp.		●	●	●			
*Ircinia* sp.			●	●		II	
*Petrosia (Petrosia) ficiformis*	(Poiret, 1789)	●	●	●			
*Spongia (Spongia) agaricina*	Pallas, 1766		●		III	III	
*Spongia (Spongia) lamella*	(Schulze, 1879)		●	●		III	EN
*Spongia* sp.		●	●	●			
Suberitidae		●	●				
**Cnidaria**							
*Eunicella cavolini*	(Koch, 1887)			●			NT
*Paramuricea clavata*	(Risso, 1826)		●	●			VU
*Parazoanthus axinellae*	(Schmidt, 1862)	●	●	●			LC
*Savalia savaglia*	(Bertoloni, 1819)		●	●	II	II	NT
Unidentified Hydrozoa		●	●	●			
**Mollusca**							
Facelinidae		●					
*Felimare picta*	(Philippi, 1836)	●					
*Octopus vulgaris*	Cuvier, 1797	●		●			
*Ostrea edulis*	Linnaeus, 1758	●		●			
*Peltodoris atromaculata*	Bergh, 1880						
*Phyllidia flava*	Aradas, 1847	●	●				
*Pleurobranchus testudinarius*	Cantraine, 1835	●					
*Pteria hirundo*	(Linnaeus, 1758)	●		●			
**Annelida**							
*Filograna* sp.		●	●	●			
*Protula tubularia*	(Montagu, 1803)	●	●	●			
*Sabella spallanzanii*	(Gmelin, 1791)		●	●			
*Serpula vermicularis*	Linnaeus, 1767	●	●	●			
**Crustacea**							
*Homarus gammarus*	(Linnaeus, 1758)	●			III	III	LC
*Munida* sp.		●					
*Palinurus elephas*	(Fabricius, 1787)	●	●	●	III	III	VU
*Stenopus spinosus*	Risso, 1827 in [Risso, 1826–1827]		●	●			
**Bryozoa**							
Encrusting bryozoa sp.1		●	●	●			
Encrusting bryozoa sp.2		●	●	●			
*Myriapora truncata*	(Pallas, 1766)	●	●	●			
*Pentapora fascialis*	(Pallas, 1766)	●	●				
**Echinodermata**							
*Peltaster placenta*	(Müller & Troschel, 1842)		●				
Centrostephanus longispinus^*^	(Philippi, 1845)	●	●		II	II	
*Echinaster (Echinaster) sepositus*	(Retzius, 1783)	●					
*Holothuria (Roweothuria) poli*	Delle Chiaje, 1824	●					LC
*Holothuria (Holothuria) tubulosa*	Gmelin, 1791	●	●				LC
*Marthasterias glacialis*	(Linnaeus, 1758)		●				
*Ophidiaster ophidianus*	(Lamarck, 1816)	●			II	II	
**Tunicata**							
*Clavelina lepadiformis*	(Müller, 1776)	●					
Didemnidae		●	●	●			
*Halocynthia papillosa*	(Linnaeus, 1767)	●	●	●			
**Pisces**							
*Anthias anthias*	(Linnaeus, 1758)	●	●	●			LC
*Apogon imberbis*	(Linnaeus, 1758)	●	●				LC
*Boops boops*	(Linnaeus, 1758)	●	●	●			LC
*Conger conger*	(Linnaeus, 1758)	●	●	●			LC
*Coris julis*	(Linnaeus, 1758)	●	●	●			LC
*Dentex dentex*	(Linnaeus, 1758)	●	●	●			VU
*Diplodus sargus*	(Linnaeus, 1758)	●	●	●			LC
*Epinephelus marginatus*	(Lowe, 1834)		●	●	III	III	VU
*Labrus mixtus*	Linnaeus, 1758		●				LC
*Muraena helena*	Linnaeus, 1758	●	●	●			LC
*Oblada melanura*	(Linnaeus, 1758)	●	●	●			LC
*Phycis phycis*	(Linnaeus, 1766)		●	●			LC
*Sciaena umbra*	Linnaeus, 1758	●	●	●	III	III	NT
*Scorpaena scrofa*	Linnaeus, 1758	●	●	●			LC
*Seriola dumerili*	(Risso, 1810)	●	●				LC
*Serranus cabrilla*	(Linnaeus, 1758)	●	●	●			LC
*Serranus scriba*	(Linnaeus, 1758)	●	●	●			LC
*Sphyraena viridensis*	Cuvier, 1829	●					LC
*Spicara smaris*	(Linnaeus, 1758)	●	●	●			LC
*Spondyliosoma cantharus*	(Linnaeus, 1758)	●	●	●			LC
*Symphodus* sp.		●					LC
*Thunnus thynnus*	(Linnaeus, 1758)		●				EN

B: Bern Convention (Convention on the Conservation of European Wildlife and Natural Habitats: Appendix II and III); S: SPA/BD Protocol (Protocol for Specially Protected Areas and Biological Diversity in the Mediterranean, Barcelona Convention: Annex II and III); I: IUCN Red List (LC: least concern; NT: near threatened; VU: vulnerable; EN: endangered); ^*^ Species included in the EU Habitat Directive.

A remarkable presence of species of commercial value was detected, both sedentary (e.g. the lobsters *Palinurus elephas* and *Homarus gammarus*, the grouper *Epinephelus marginatus* and the brown meagre *Sciaena umbra*) and migratory (e.g. the greater amberjack *Seriola dumerili* and the bluefin tuna *Thunnus thynnus*) (Fig. 6c,g,i–j). The latter ones were swimming close to the coral forest as both big solitary specimens and large groups searching for prey. Both males and females of sexually-dimorphic species such as the cuckoo wrasse *Labrus mixtus* (Fig. 6d) were observed with their typical reproductive livery, providing further evidence of the coral forest as breeding and spawning area.

### Modelling the marine currents

**Figure 7:**
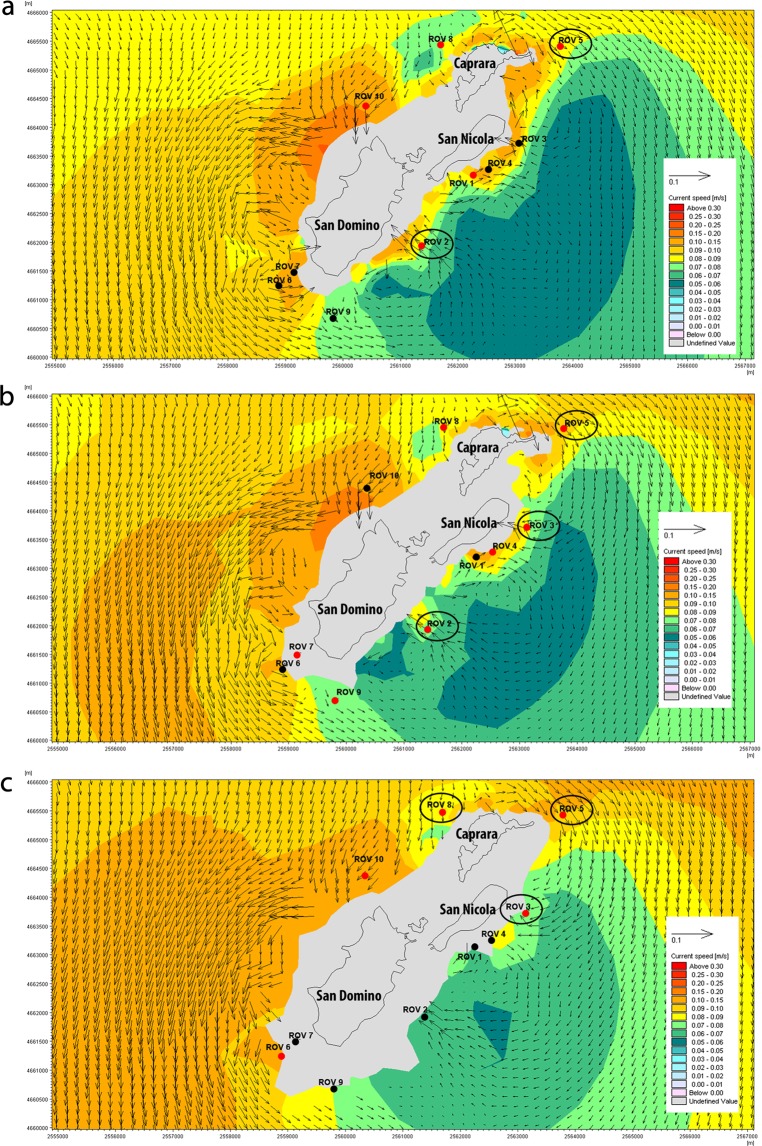
Mean surface currents around Tremiti Islands at the depths of: (**a**) 50 m, (**b**) 60 m and (**c**) 70 m, with localization of the sites explored using the ROV. Red dots: hard bottom; black dots: soft bottom; black circles: sites where *Antipathella subpinnata* was observed. Map has been created using MIKE ZERO powered by DHI (https://www.mikepoweredbydhi.com).

Modelling confirmed the dominance of cold, oxygenated and trophic-carrying water masses in the Adriatic Sea, coming from the north and proceeding southwards^20,21^, and strong enough to support suspension feeders such as corals. In particular, water masses from 50 to 70 m depth hit the Tremiti Archipelago on the north-northwest coast of Caprara Island, in proximity of the site 8 (Fig. 7a–c). Then, water masses split in two, flowing eastwards along the coast of Caprara and south-westwards along San Domino Island. The first mass proceeds anticyclonically, passing through the sites 5 and 3, thus probably connecting the three *A. subpinnata* sub-populations studied. From site 5, the water mass proceeds southwards and part of it turns anticyclonic, skips the coast of San Nicola Island and reaches the southeast coast of San Domino, in the proximity of the site 2, most likely determining the presence of few colonies of *A. subpinnata* in this site.

The second water mass that, hitting the Caprara Island from the north, proceeds south-westwards along the San Domino Island, does not pass through site 8 and probably does not carry propagules on the west side of the archipelago. Although currents are strong enough to support black coral communities, *A. subpinnata* was not present all along the path of this second water mass, most of which create a strong cyclonic vortex that proceeds southwards (Fig. 7a–c).

Currents resulted fundamental in shaping the distribution of *A. subpinnata* in presence of hard substrata and a minimum water speed of 0.08 m s^−1^ seemed to be needed for the survival of these corals. On the contrary, both mean and maximum water temperatures were comparable all over the explored sites and did not allow to explain the absence of *A. subpinnata* from some of them (Figs. S1–S2).

### Impacts and threats

**Figure 8:**
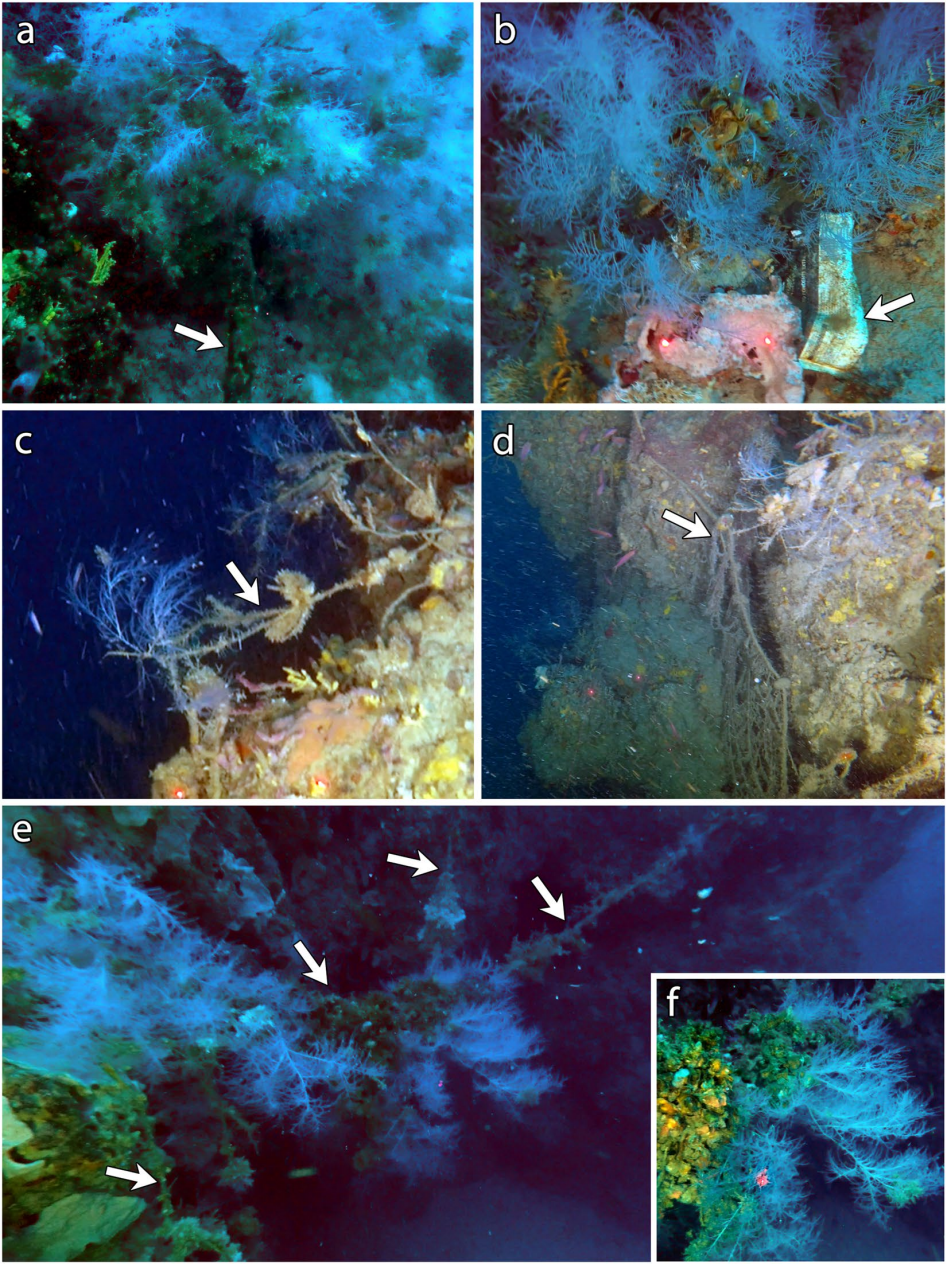
Impacts. *Antipathella subpinnata* with: (**a**) steel cable (arrow); (**b**) plastic litter (arrow); (**c**) longline (arrow); (**d**) gillnet (arrow); (**e**) tangle of longlines (arrows) colonized by clusters of oysters and colonies of *A. subpinnata*; (**f**) detail of the colonized longline.

Lost fishing gears were the most common anthropic impact detected within the forest of *A. subpinnata*. In particular, two steel cables, one of which directly within a group of *A. subpinnata* colonies, were observed on coralligenous outcrops at site 2, where only a few colonies were detected (Fig. 8a). These cables were colonized by encrusting fauna and have probably been lost several years ago due to trawling operation too close to the hard bottom. Two lost longlines and few plastic objects were observed in the proximity of the colonies at site 3 (Fig. 8b), while 5 nets and 39 longlines were observed within the coral forest at site 5, where lost fishing gears showed a density of 0.14 ± 0.26 items m^−2^. These gears were entangled on the coralligenous outcrops as well as on the colonies of *A. subpinnata* and *P. clavata* but, in some cases, several specimens of *O. edulis* and colonies of *A. subpinnata* were settled on them, using the lost gears as a substratum (Fig. 8e–f). Furthermore, 5 longlines and 8 nets were observed within the mixed coral forest at site 8 (Fig. 8c–d). All these lost gears, some colonized by fauna and others clearly recent, proved the presence of past and present destructive fishing practices carried out in the proximity of the *A. subpinnata* forest, including in the highly protected zone (Zone B; sites 5 and 8) of the MPA.

## Discussion

Marine explorations all over the Mediterranean Sea are revealing an exponential amount of coral communities from the mesophotic to the aphotic zones, highlighting how these extensive environments are still scantly explored, even in a well-studied basin as the Mediterranean^5,22^. Although Tremiti Islands are an MPA since 1989, the seabed below 50 m depth was completely unexplored and the presence of *A. subpinnata* was overall unknown. This study revealed the presence of a dense forest of *A. subpinnata* mostly at the borders of the MPA (Fig. 1). The extraordinary colonies density was overall higher than the one of the forests studied thus far, such as those in the Tyrrhenian Sea^7,9^, in the Sardinia Channel^14^ and in the Azores^11^, as well as those of the congeneric *Antipathella fiordensis* (Grange, 1990) studied in the southern fiords of New Zealand^23^. Moreover, the population of the Tremiti Islands represents one of the few monospecific forests of *A. subpinnata* known and the only record of a forest settled on vertical substrata^8,9^.

The asymmetrical size structure of the population, with significant positive values of skewness, reflected the dominance of small-size colonies, as well as the concomitant leptokurtic distribution (mesokurtic at site scale) indicated a more peaked distribution than a normal one, with longer tails. These results were in accordance with those found for the forests of both *A. subpinnata* in the Mediterranean Sea^9^ and *A. fiordensis* in New Zealand^23^, with the general dominance of colonies up to 40–60 cm high. Small sizes can indicate young, sexually-immature colonies^17^, or can be due to fragmentations as a control mechanism of the colony’s growth, generally in response to strong currents or as a consequence of both natural and anthropic stresses, as observed with other anthozoans (e.g^24–27^.). In the case of *A. subpinnata*, fragmentation has been recently described as an important propagation strategy^28^. At Tremiti Islands, this process is unlike to be relevant in sites where there are large isolated colonies (site 8) and on vertical substrata (site 5), while it can contribute to the large density of the sub-population settled on the top of the shoals (site 3 and part of site 5).

Relatively small colony sizes might also be due to the presence of fishing impacts. In fact, the site 5 was the only one lacking colonies higher than 80 cm (Fig. 4b), but also the one with the highest number of lost fishing gears.

### Epibionts and associated megafauna

The colonies of *A. subpinnata* can host several species of epibionts, generally settled on the branches^8^. Epibiont fauna was mostly comparable to that found by other authors^9^, with the large presence of *Filograna* spp., bryozoans and ascidians, but, in our case, also *O. edulis* and *P. hirundo* were relevant in the epibiont community (Fig. 5). Moreover, the presence of brown macroalgae on some colonies was reported here for the first time and could be an alarming signal for the future, as their fast growth rate could affect the survival of the *A. subpinnata* colonies at their shallowest limit (50–60 m depth) as well as that of *P. clavata* and other habitat formers.

The forest of *P. clavata* from 30 to 50–60 m depth and that of *A. subpinnata* down to 80 m depth create a hotspot of biodiversity that attracts vagile fauna, including species of both conservation interest and high commercial value. Although the ROV allowed to recognize only a small number of megafauna taxa and it was not the elective tool for the assessment of vagile fauna, the high number of fish species encountered indicated the importance of coral forests in terms of associated community. The community richness might be enhanced by the presence of other benthic habitat formers, as the bioconstructors of the coralligenous (i.e. serpulids, bryozoans, molluscs, sponges and other corals)^19^ as well as the patches of *P. rubra* on the nearby soft bottom^29,30^.

### Fishing impacts

Lobsters, groupers and pelagic predators often frequent the coral forests, that are consequently targeted by fishing practices in spite of the protection regime and of the high possibility to lose the gears because of the rough topography of the area. Paradoxically, the two sites with the highest number of lost fishing gears (site 5 and site 8) were present in the B Zone of the MPA, such as the highly protected area. Although these sites are not feasible for trawling, other fishing practices based on the use of fixed gears such as longlines, trammel nets and gillnets can be carried out very close to the coralligenous habitat and the steep rocky walls. These gears are considered fixed and less invasive compared to trawl nets but, although static on the seabed, they may be pulled across the bottom for short distances by strong currents or during retrieval operations. For this reason, they represent destructive fishing gears for three-dimensional benthic organisms, showing a significant mechanical impact on VME indicator taxa such as corals, and their use should be banned from areas characterized by coral forests^5^.

### Corals and currents

Strong currents play a crucial role for the survival of black corals, carrying food particles and supplying organic matter^31^. The water masses coming from the north Adriatic Sea are cold and oxygenated due to the cold winds, while the Po river guarantees the nutrient supply^32^. These currents are fundamental for the survival of the black coral forest of Tremiti Islands and sustain rich cold-water coral communities present southwards, in the aphotic zone of the Apulian plateau, like those of the Bari Canyon and the Santa Maria di Leuca coral provinces^6,22,32,33^. The distribution of *A. subpinnata* at Tremiti Archipelago seemed to be particularly linked to the path of the north-coming water masses that hit the Caprara Island and proceed eastwards (Fig. 7a–c). The population of site 8 was likely to be the primary parental situation in shedding eggs and propagules along the archipelago. This was also the site where the biggest colonies where found, probably representing the main ones involved in sexual reproduction. The north-coming currents were likely to connect all the sites where corals were found, while the nearby site 4 (ca. 700 m far) was mainly interested by an anticyclonic water mass of different origin, thus justifying the absence of *A. subpinnata* even in presence of strong currents and hard substrata. Currents from site 2 hit the coast and were probably channelled among the main islands, where the depth shallower than 50 m was not feasible for the survival of *A. subpinnata* due to temperature variations subjected to seasonal cycles^8^.

## Conclusions

The *A. subpinnata* forest of Tremiti Islands is one of the biggest known thus far in the Mediterranean Sea, the only one in the Adriatic Sea and the only one settled on a vertical substrate, representing a unique VME in the basin. It is a key area for the conservation and sustainable management of species of commercial interest that use the coral forest as both refuge and feeding area as well as, in some cases, as a spawning area. Further zoological studies are needed, with *ad hoc* sampling of colonies’ branches to deepen in the histology and genetic of the species, in order to assess the sexual maturity and the eventual genetic connectivity of the colonies within the Tremiti population and with other populations of the Mediterranean Sea.

Destructive fishing practices targeting valuable commercial species such as lobsters, groupers and big pelagic fish are present in the area characterized by the black coral forest, particularly in the highly protected zone of the MPA. The majority of the fishing gears observed was recent, attesting a non-negligible impact of fixed fishing gears such as longlines, gillnets and trammel nets. Although regulated through fishing licenses, the former management of the Zone B is not effective in the protection of VMEs such as black coral forests. Destructive practices, such as fishing and anchoring that can severely threat the survival of arborescent corals, should be banned in the proximity of the coral forests. Besides the direct removal of the colonies, these practices can severely damage the coenenchyme of the corals and may also enhance infections as well as the susceptibility to epibiosis. Therefore, a revision of the MPA borders and zonation is urgently needed to extend the MPA limit at 100 m depth and to include the forest of *A. subpinnata* in a no-fishing area. Considering that illegal fishing practices are likely to occur, the enforcement of regulations and controls to avoid every type of fishing activity in proximity of the *A. subpinnata* forest is urgently needed, as regulations of fishing activities in extremely sensitive habitats as coral forests are not sustainable.

## Materials and methods

### Study area

The study area is represented by Tremiti Islands MPA (42°7.38′N − 15°30.02′E) (Fig. 1), located in the Adriatic Sea, 12 nautical miles north of the Gargano promontory (Apulia Region, Southern Italy). The MPA involves an archipelago which consists of three main islands called San Domino, San Nicola and Caprara, a smaller island called Cretaccio, and a further island called Pianosa located 12 nautical miles north-east the three main islands. The area is characterized by a high aesthetic value due to the diversity of natural landscapes and cultural evidences^34^, including a Site of Community Importance, a Special Protected Area and an Important Bird Area. The MPA shows a gradient of restrictions and is divided into three main zones: Zone A (no take, no entry zone) confined to the uninhabited island of Pianosa, where all human activities are forbidden; Zone B (highly protected zone) along most of the Caprara coast and part of the San Domino coast, where bathing, sailing and free diving are allowed, scientific research, scuba diving, motor navigation and professional fishing are subjected to authorization, while anchoring and recreational fishing are forbidden; Zone C (partially protected zone) in the rest of the archipelago, where most human activities (e.g. bathing, diving, boat traffic, anchoring, recreational fishing) are allowed, while scientific research and professional fishing have to be authorized (Fig. 1). The borders of the MPA are located around the bathymetry of 70 m depth.

### Habitat mapping

Acoustic data were obtained from ship-based research surveys using Side-Scan Sonar (100/500 kHz Klein3000) and Multi-Beam Echosounder (455 kHz Reason Seabat 8125), while positional data were provided by a Hemisphere Crescent R-Series dGPS and water sound velocity was obtained using the Seabird SBE 21 device. Data were elaborated using CARIS SIPS 8 software, then imported in a GIS environment using ArcView 10.2 for the interpretation and the cartography. Mapping was supported by ground-truthing using a towed camera for punctual observations, then all the hard bottoms below 50 m depth, feasible for the settlement of black corals, were identified. Hard bottoms were mostly represented by rocky shoals and coralligenous bioconstructions, i.e. a typical mesophotic Mediterranean habitat built up by a suite of calcifying organisms (e.g. calcareous red algae, corals, serpulids, bryozoans, molluscs) that grow one on the other, generation after generation, building a secondary hard substratum^19,34^. The ten best sites for potential black coral settlement were selected for the ROV surveys (Fig. 1).

### Video recording and analyses

During September 2018, a total of 10 sites between 50 and 100 m depth were explored using the ROV *Prometeo* equipped with a 4 K video camera, a depth sensor, a compass for underwater navigation, a maximum scene illumination of 13,000 lumen, as well as two parallel laser beams that provided a fixed scale used in the subsequent video analyses. ROV transects covered an area of ca. 300–350 m^2^ each on hard bottoms.

Transects where *A. subpinnata* was observed were analysed using Adobe Premiere Pro and ImageJ software^35,36^. Sampling units of 2.5 ± 0.2 m^2^ were defined along each transect, according to the minimal area identified by^37^ based on species-area curves for sessile invertebrates in Mediterranean benthic communities and suitable for ROV imaging^38,39^. Sequences with bad visibility due to water turbidity or distance from the seabed, as well as sequences recorded outside the coral forest (e.g. soft bottoms and hard-bottom areas not colonized by *A. subpinnata*) were discarded. The forest of *A. subpinnata* was quantified both by occupancy (frequency of occurrence in the set of sampling units) and by abundance (number of colonies per sampling unit), then the density (colonies m^−2^) was calculated for each sampling unit and expressed as mean ± standard error. Epibiosis and anthropic impacts observed within the coral forest were also assessed and the associated megafauna was identified at the lowest possible taxonomic level, although a reliable quantification of most of the taxa was not possible using the ROV.

High-resolution still images for morphometric analysis were extracted directly from the ROV footage when laser-beams were in the same plane as the corals, in order to measure the height of the colonies whose position and ROV framing allowed it. Eight size classes of 20 cm each were identified, from 1–20 to 141–160 cm, according to the previous studies^9^. Size structure was assessed for those sites with more than 30 measured colonies, as populations with lower values could compromise skewness and kurtosis estimates^40^. Size structure was analyzed for each sub-population in terms of size-frequency and distribution parameters, such as skewness and kurtosis, calculated by means of the R software functions *agostino.test*^41^ and *anscombe.test*^42^. The same analysis was also carried out considering together all the colonies of *A. subpinnata* that were possible to measure in the study area (N = 202), both in the coral forest and in those sites with less than 30 colonies measured.

### Sampling and species identification

*Ad hoc* non-invasive sampling of a small portion of black coral colonies within the depth of 60 m was carried out by scuba diving in order to confirm the taxonomic identification of *A. subpinnata*^1,8^. Coral polyps were photographed *in vivo*, then terminal branches portions of 8–10 cm in length were sampled from 10 different colonies in two sites (site 2 and site 5) and preserved in ethanol. Subsamples were cleaned from the coenenchyme with gentle rinses in diluted sodium hypochlorite (NaClO), then washed with distilled water and dehydrated in a graded ethanol series samples. Dried axis was coated with gold-palladium in an Edwards S150A Sputter coater and examined with a Hitachi TM3000 Scanning Electron Microscope (SEM).

### Modelling of marine currents

The 3D hydrodynamic numerical model MIKE 3 FM HD produced by the Danish Hydraulic Institute^43^ was performed for one year starting from 01 January 2018 at 00:00 UTC in order to study a possible relationship between the sea currents and the observed presence of black corals at Tremiti Archipelago. This mathematical model solves the three-dimensional time-dependent conservation equations of mass and momentum (the Reynolds-Averaged Navier-Stokes equations) adopting the explicit finite difference method, with the Courant-Friedrichs-Lewy stability condition. The basic characteristics, numerical formulation and process equations of the model MIKE 3 FM were provided by^43^. The hydrodynamic simulation was carried out in the baroclinic model, with temperature and salinity vertical profiles extracted by the Mediterranean Sea Physics Reanalysis model (horizontal grid resolution of 1/16° and 72 unevenly spaced vertical levels) with the aims of improving the numerical approach and model more realistic conditions. Moreover, the simulation was forced at the sea open boundary by the u-v velocities vertical profiles extracted by the Mediterranean Sea Physics Reanalysis model.

The atmospheric data spacing varying and available every 6 h (u and v components of wind [m/s], atmosphere pressure [Pa], total cloud cover [%], solar radiation [J/m^−2^], air temperature [°C]) were extracted by ERA5 developed through the Copernicus Climate Change Service (C3S). The precipitation data [mm/d] spacing varying and available every 1-day was predicted by CPC Merged Analysis of Precipitation.

The turbulent closure model used within MIKE 3 FM HD model relied on the k-ε formulation for the vertical direction^44^ and the Smagorinsky formulation for the horizontal direction^45^. The Smagorinsky coefficient was assumed uniform in space and temporally constant, equal to 0.6. According to the sensitivity analysis shown in another Mediterranean area^46^, the simulation was performed by adopting a seabed roughness equal to 0.1 m. The wind drag coefficient *C*_*d*_ was considered as the calibration parameter to which the model results were most sensitive, based on recent studies^47–50^. The simulation was performed by adopting the wind drag coefficient *C*_*d*_ equal to 0.02, according to^51–53^.

## Supplementary information

Supplementary information.

## Data Availability

The datasets generated during and/or analysed for the current study are available from the corresponding author upon request.
