# Supplementary material for: A mesophotic black coral forest in the Adriatic Sea
Source: Sci Rep. 2020 May 22;10:8504. doi: 10.1038/s41598-020-65266-9 (PMC7244545; doi:10.1038/s41598-020-65266-9)
Supplement: Supplementary file 1 — Supplementary information. [file 41598_2020_65266_MOESM1_ESM.docx]

**A mesophotic black coral forest in the Adriatic Sea**

Giovanni Chimienti^1,2*^, Diana De Padova^3^, Michele Mossa^3^, Francesco Mastrototaro^1,2^

^1^Department of Biology, University of Bari Aldo Moro, Bari, Italy

^2^CoNISMa, Roma, Italy

^3^Polytechnic University of Bari, DICATECh, Bari, Italy

*Correspondence: G. Chimienti, Department of Biology and CoNISMa LRU, University of Bari Aldo Moro, Via Orabona 4, 70125 Bari, Italy. Tel: +39 080 5443330. E-mail: [giovanni.chimienti@uniba.it](mailto:giovanni.chimienti@uniba.it)

| **Site** | **Depth (m)** | **Start** | **End** | **N** | **Habitat** |
| --- | --- | --- | --- | --- | --- |
| **1** | 55–65 | 42° 07.657’ N 15° 30.131’ E | 42° 07.207’ N 15° 30.697’ E | 0 | Detritic seabed with patches of rhodoliths and small, scattered coralligenous outcrops. |
| **2** | 50–75 | 42° 06.498’ N 15° 30.079’ E | 42° 06.498’ N 15° 30.079’ E | 9 | Coralligenous dominated by a forest of *P. clavata* with large colonies (> 1 m high) on both horizontal and vertical substratum, and few isolated colonies of *A. subpinnata* on sub-horizontal hard bottom at 52–62 m depth. |
| **3** | 62–67 | 42° 07.462’ N 15° 31.275’ E | 42° 07.502’ N 15° 31.376’ E | 387 | Scattered rocky formations with coralligenous bioconstructions, some of which with few isolated colonies of *A. subpinnata* (62-66 m depth) and others with large colony aggregations on top (62-64 m depth). |
| **4** | 58–65 | 42° 07.250’ N 15° 30.889’ E | 42° 07.180’ N 15° 30.818’ E | 0 | Coralligenous dominated by sponges and ascidians. |
| **5** | 30–80 | 42° 08.346’ N 15° 31.713’ E | 42° 08.396’ N 15° 31.814’ E | 379 | Coralligenous on both horizontal and vertical bottom with a monospecific forest of *P. clavata* at 32–50 m depth, a mixed aggregation of *P. clavata* and *A. subpinnata* at 51–60 m depth, and an almost monospecific forest of *A. subpinnata* at 60–80 m depth on vertical substratum. |
| **6** | 70–80 | 42° 06.139’ N 15° 28.236’ E | 42° 06.177’ N 15° 28.339’ E | 0 | Small, scattered coralligenous outcrops on muddy bottom dominated by sponges and bryozoans. |
| **7** | 55–64 | 42° 06.251’ N 15° 28.375’ E | 42° 06.330’ N 15° 28.313’ E | 0 | Coralligenous dominated by *P. clavata* from 60 to 63 m depth. |
| **8** | 53–84 | 42° 08.354’ N 15° 30.264’ E | 42° 08.432’ N 15° 30.296’ E | 64 | Shoal with coralligenous patchy dominated by *P. clavata*, with isolated colonies or small patches of *A. subpinnata* at 68–77 m depth on both the vertical wall and the sub-horizontal niches, often in mixed aggregation with *P. clavata*. |
| **9** | 55–72 | 42° 05.784’ N 15° 28.909’ E | 42° 05.870’ N 15° 28.864’ E | 0 | Coralligenous dominated by *E. cavolini* at 55–60 m depth, followed by small coralligenous outcrops on muddy/detritic seabed. |
| **10** | 50–80 | 42° 07.381’ N 15° 29.281’ E | 42° 07.741’ N 15° 29.301’ E | 0 | Detritic seabed with patches of rhodoliths and small, scattered coralligenous outcrops. |

**Table S1.** Geographic coordinates and depth range of the ten ROV transects, with indication of the habitat type and the number of *A. subpinnata* colonies observed (N).


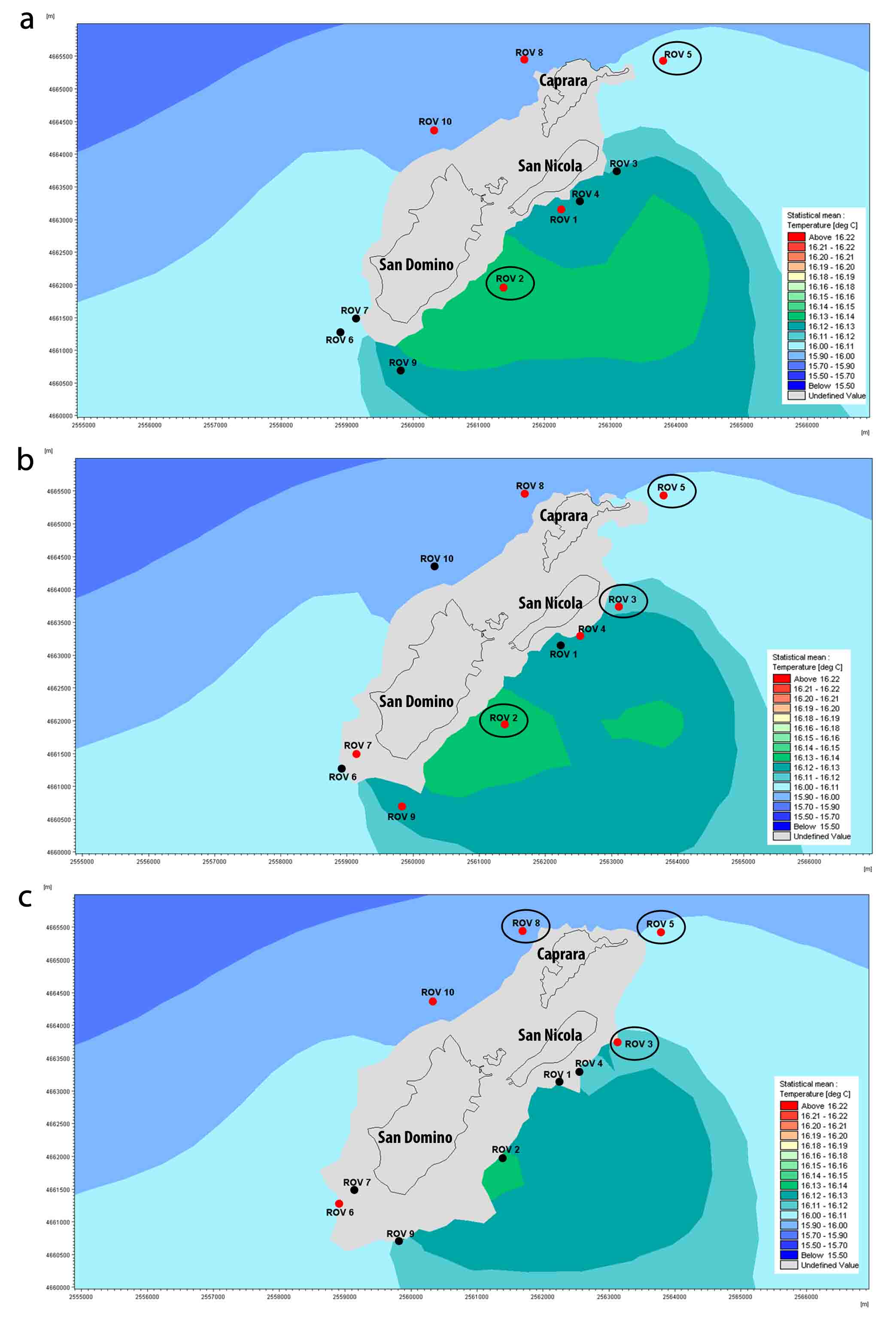


**Figure S1.** Mean annual temperature in the study area at the depths of a) 50 m, b) 60 m and c) 70 m. Red dots: hard bottom; black dots: soft bottom; black circles: sites where *Antipathella subpinnata* was observed. Map has been created using MIKE ZERO powered by DHI (https://www.mikepoweredbydhi.com).


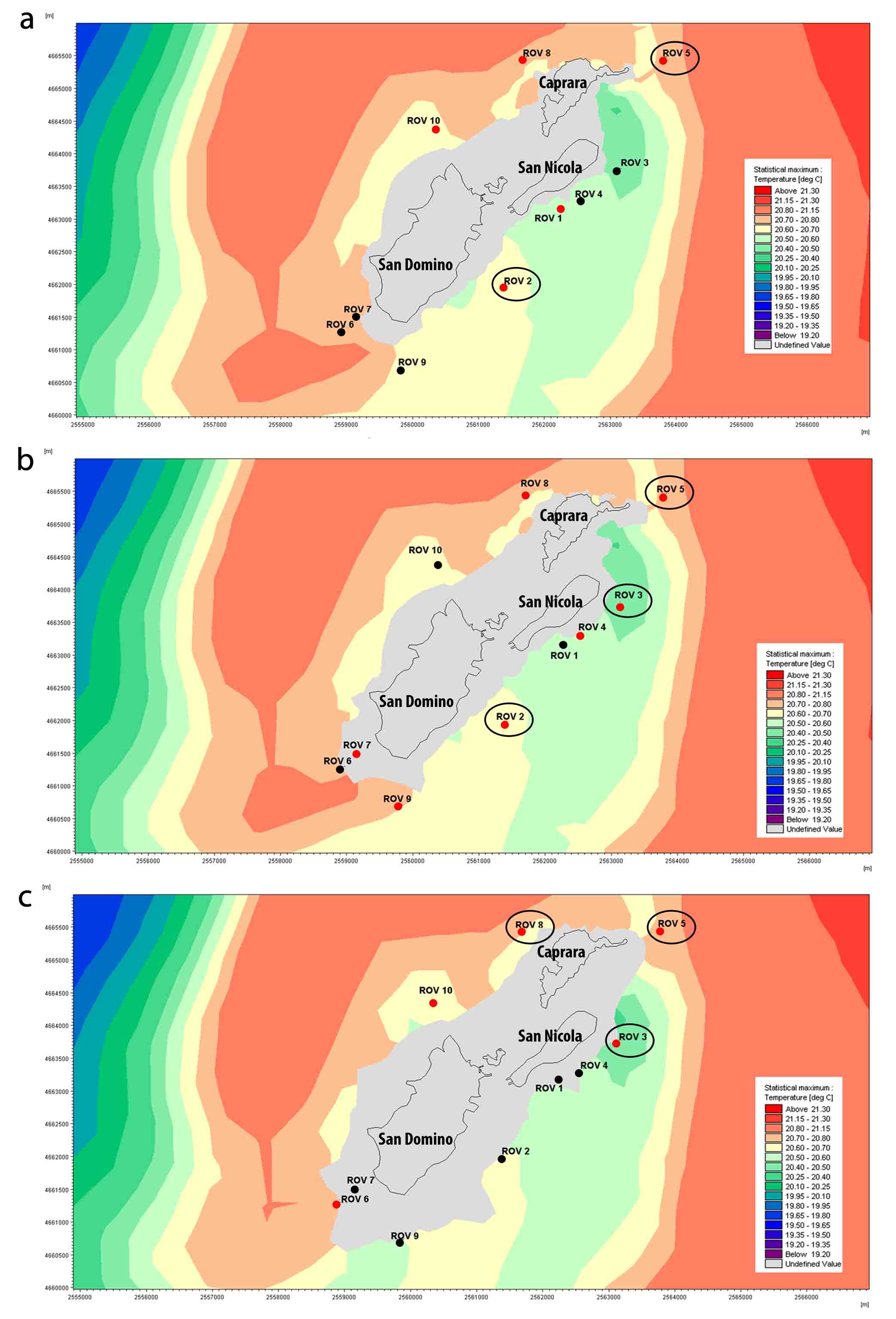


**Figure S2.** Maximum annual temperature in the study area at the depths of a) 50 m, b) 60 m and c) 70 m. Red dots: hard bottom; black dots: soft bottom; black circles: sites where *Antipathella subpinnata* was observed. Map has been created using MIKE ZERO powered by DHI (https://www.mikepoweredbydhi.com).
